# Mental states via possessive predication: the grammar of possessive experiencer complex predicates in Persian

**DOI:** 10.1007/s11050-024-09221-8

**Published:** 2024-04-05

**Authors:** Ryan Walter Smith

**Affiliations:** Department of Linguistics and English Language, The University of Manchester, Manchester, UK

**Keywords:** Mental states, Property concept sentences, Possession, Complex predicates, Persian, Gradability

## Abstract

Persian possesses a number of stative complex predicates with *dâshtan* ‘to have’ that express certain kinds of mental state. I propose that these *possessive experiencer complex predicates* be given a formal semantic treatment involving possession of a portion of an abstract quality by an individual, as in the analysis of property concept lexemes due to Francez and Koontz-Garboden (Language 91(3):533–563, 2015; Natural Language and Linguistic Theory 34:93–106, 2016; Semantics and morphosyntactic variation: Qualities and the grammar of property concepts, Oxford University Press, 2017). Augmented with an analysis of prepositional phrases introducing the target of the mental state and an approach to gradability in terms of measure functions (Wellwood in Measuring predicates, PhD dissertation, University of Maryland, College Park, 2014), the analysis explains various properties of possessive experiencer complex predicates, including the behavior of target phrases, the ability of the non-verbal element to be modified by a range of adjectives, the direct participation of the non-verbal element in comparative constructions, and the ability of degree expressions to modify both the non-verbal element and the VP containing the complex predicate. Theoretically, the analysis ties transitive mental state expressions to the grammar and semantics of property concept sentences, which are expressed via possessive morphosyntax cross-linguistically, and connects with syntactic proposals that independently argue for a universal underlyingly possessive morphosyntax for mental state predicates (Noonan in Case and syntactic geometry, PhD dissertation, McGill University, 1992; Hale and Keyser in Prolegomenon to a theory of argument structure, MIT Press, 2002). The work here also motivates modifications to Francez and Koontz-Garboden’s original proposal, and opens new questions in the original empirical domain of the analysis of possessive predicating strategies for the expression of property concept sentences.

## Introduction

It is common cross-linguistically for *property concept sentences*, the translational equivalents of English predicative adjectival sentences, to be expressed morphosyntactically via possession of an abstract noun (Dixon 1982, Baglini 2015, Francez and Koontz-Garboden 2015, 2017, Hanink et al. 2019). Such *possessive predicating* strategies for the expression of property concept sentences have been noted and analyzed in a variety of languages, such as Ulwa (Koontz-Garboden and Francez 2010; Francez and Koontz-Garboden 2017), Hausa (Newman 2000; Francez and Koontz-Garboden 2017), Wolof (Baglini 2015), Basaá (Hanink et al. 2019), and Washo (Hanink and Koontz-Garboden 2021), among others. To give just one example, the majority of property concept sentences in Hausa are formed through a combination of the preposition *da* ‘with’ with an abstract noun of sensory quality, such as *karfi* ‘strength,’ yielding sentences like (1) to express the proposition ‘we are strong.’ Crucially, *da* is also used in the language to express possession more generally, hence its designation as a language with a possessive predicating strategy for the expression of property concept sentences.

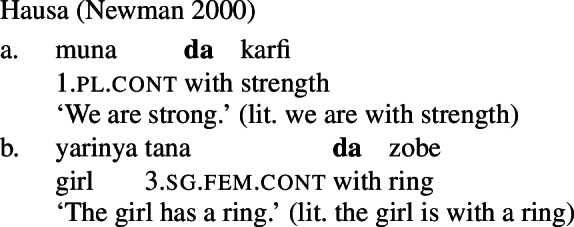

Previous work on possessive predication in the grammar of property concept sentences has focused on the translational equivalents of adjectives, as many languages with a small, closed (or non-existent) class of adjectives make use of the possessive predicating strategy (Dixon 1982; Francez and Koontz-Garboden 2017). However, one can ask similar questions about the expression of properties by lexical categories other than adjectives. For instance, Baglini (2015) and Francez and Koontz-Garboden (2017) point out that the grammar of Wolof groups possessive predicating property concept lexemes with non-possessive predicating *stative verbs*, both intransitive and transitive. The authors of these works conclude that both types of expression have denotations built from the same ontology. We thus might expect to find languages with possessive predicating strategies for the expression of transitive statives.

We don’t have to go far to find possessive paraphrases for transitive statives: even in English such paraphrases are possible, and it is not uncommon to speak of having faith in someone or having an interest in something. This said, the possessive strategy is often very marked and unidiomatic, especially when the mental state noun is unmodified, and in some cases is very odd.

(2)
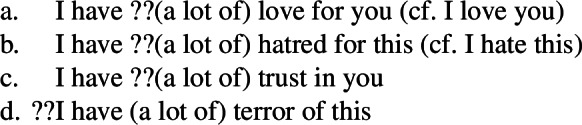

A better testing ground for investigating the properties of possessive predicating mental state predicates would be a language in which a possessive strategy is the primary means for expressing such predicates.^1^

In this paper, I examine the use of possessive predication in Persian to express a range of *mental states*, serving as the translational equivalents of transitive stative verbs in English. Persian, a language with a small, closed class of verbs, is an ideal language to investigate the behavior of possessive predicating strategies for the expression of mental state predicates, because the majority of mental state predicates are expressed via a combination of the possessive verb *dâshtan* and an abstract noun naming the particular mental state. For example, the translational equivalents of the English stative verbs *love* and *hate* are expressed by *eshgh dâshtan* and *nefrat dâshtan*, literally ‘to have love’ and ‘to have hatred,’ respectively, as in (3).

(3)
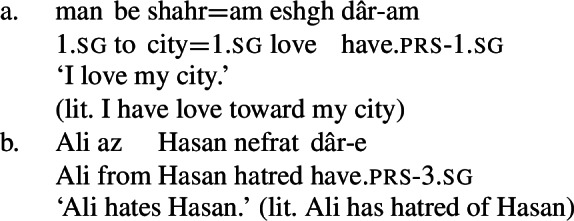

I propose that these *possessive experiencer complex predicates* be analyzed as involving possession of a portion of an abstract quality by an individual, as in Francez and Koontz-Garboden’s (2015, 2016, 2017) analysis of property concept sentences. Augmented with an analysis of prepositional phrases introducing the target of the mental state and an approach to gradability in terms of measure functions (Wellwood 2014), the analysis explains various properties of possessive experiencer complex predicates, including the distribution of target phrases in and outside of complex predicates, the ability of the possessed nominal to be modified by a range of adjectives, the direct participation of the nominal in comparative constructions, and the ability of degree morphemes and other modifiers to attach at both the nominal and VP level with the same meaning.

Theoretically, the analysis ties mental state predicates to the grammar and semantics of property concept sentences: mental state nominals are property concept lexemes describing human propensities in Dixon’s (1982) sense, and likewise pattern with other quality nouns not only in Persian, but also in other languages, including English. Moreover, possessive predicating strategies for mental state predicates exist in languages, other than Persian, such as Sorani Kurdish and Irish, suggesting a broader cross-linguistic applicability of the proposal. Finally, an adequate account of the phenomena reported here motivates modifications to Francez and Koontz-Garboden’s original proposal for the analysis of property concept sentences, and opens new questions in the original empirical domain of possessive predication in such sentences, particularly concerning the extent of the modifiability and compositional flexibility of quality-denoting nominals.

The paper is structured as follows. Section 2 contains background on the Persian language, including a discussion of complex predicates. Section 3 introduces the primary empirical focus of the paper, what I call possessive experiencer complex predicates, which make use of a possessive-predicating strategy for expressing a variety of mental state predicates, and details some of their noteworthy properties. Section 4 introduces Francez and Koontz-Garboden’s analysis of property concept sentences in terms of possession of portions of qualities, shows that the NVEs of possessive experiencer complex predicates behave like quality nouns, and develops a preliminary analysis of possessive experiencer complex predicates, staying as close to Francez and Koontz-Garboden’s assumptions as possible. In so doing, the section also demonstrates problems that arise with this initial analysis. Section 5 proposes revisions to Francez and Koontz-Garboden’s analysis, and shows how the new analysis overcomes the problems with the preliminary analysis in Sect. 4, and details additional correct predictions. Section 6 considers the implications of the proposal beyond Persian by providing evidence for the existence of possessive predicating strategies for the expression of mental state predicates in languages other than Persian, with analogous phenomena occurring in Sorani Kurdish and the Celtic languages Irish and Scottish Gaelic, and considers an analysis of English-like mental state verbs constructed from the ingredients of the analysis of the Persian phenomena discussed throughout the paper. Section 7 concludes the paper by taking stock and discussing avenues for future research.

## Background on Persian

Persian (also known as *Farsi*) is a southwestern Iranian language (Indo-European), spoken in Iran, Afghanistan, and Tajikistan as an official language. It exhibits thorough nominative-accusative alignment with differential object marking of specific NPs. The basic word order is SOV, but is otherwise head-initial: the language has prepositions rather than postpositions, nouns precede adjectives in the NP, and embedded clausal complements follow the head verb or noun. Verbs have distinct past and present tense stems. The past tense stem is predictable from the infinitive (*didan* ‘to see’ → *did* ‘saw (3.sg)’) but the present stem is usually unpredictable (***did****am* ‘I saw,’ but *mi****bin****am* ‘I see’). (4), (5), and (6) illustrate a few of these properties.

(4)

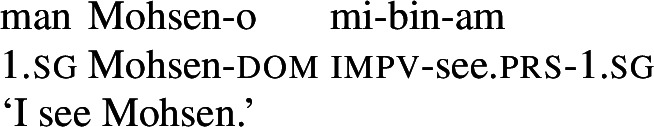

(5)

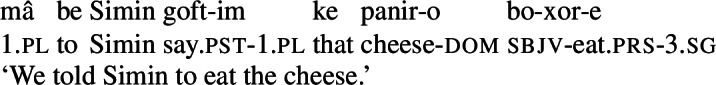

(6)
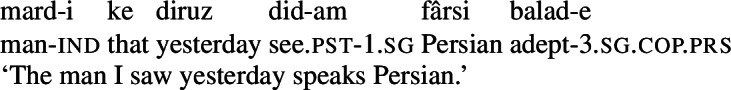

Persian has several distinct varieties, partially depending on which country it is spoken in: the main varieties are Iranian Persian, Afghan Persian (Dari), and Tajiki Persian (Tajik) (Karimi 2005; Jasbi 2015).^2^ Moreover, Persian is diglossic: many differences exist between the formal language and the colloquial language, and include differences in agreement suffixes of verbs, nominal suffixes, and systematic differences in pronunciation (Jasbi 2015). The Persian sentences and judgments reported in this paper, unless otherwise noted, come from elicitation sessions conducted by the author with three native speakers of colloquial Iranian Persian, and it is this variety that I refer to simply as *Persian* throughout this paper.

### Possession and the *ezâfe* construction

Persian has a possessive verb, *dâshtan* ‘to have.’ It is mostly used the way ‘have’ is used in English, namely, to express possession, whether alienable or inalienable.^3^

(7)
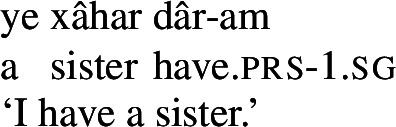

(8)

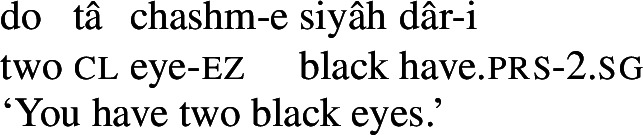

(9)
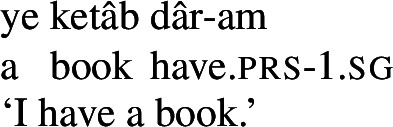

For possession within the noun phrase, Persian makes use of the *ezâfe* (lit. addition) construction. The *ezâfe* morpheme surfaces as -*e* after consonants and -*ye* after vowels. *Ezâfe* is insensitive to the distinction between alienable and inalienable possession: an *ezâfe* morpheme is obligatory regardless.

(10)

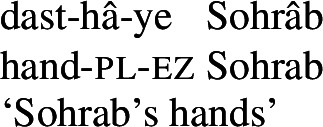

(11)
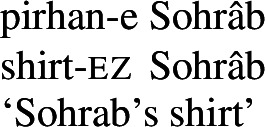

*Ezâfe* is used for possession within the NP and more generally for modification of a noun by adjectives as well as by other nouns. If there are multiple adjectives or nouns within an NP, an *ezâfe* vowel appears between each one.

(12)

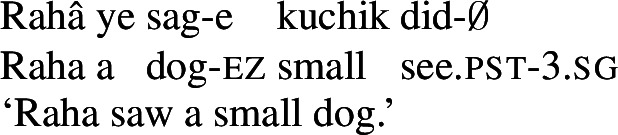

(13)

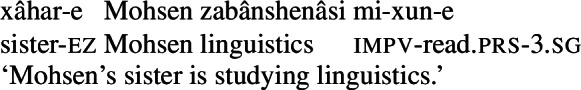

(14)
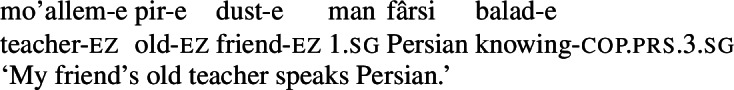

Several analyses exist of the *ezâfe* construction, ranging from treating it as a marker of Case on +N elements (Samiian 1994; Larson and Samiian 2020), a marker of agreement triggered after roll-up movement (Kahnemuyipour 2014), and a linker affixed to heads at PF (Ghomeshi 1997). I take no stance on the correct analysis of *ezâfe*, and though I generally take it to be semantically vacuous, nothing hinges on this choice. My goal is simply to flag its existence and describe its properties for readers not familiar with Persian, as *ezâfe* appears in examples featuring nominal modification throughout this paper.

### Complex predicates

While Persian possesses an open class of nouns and adjectives, verbs form a closed class: there are only around 115 simplex verbs in the language (Khanlari 1973; Mohammad and Karimi 1992), and new members cannot be freely added to the category. To compensate for its relatively small inventory of verbs, Persian makes heavy use of complex predicates, also known as *light verb constructions* and *compound verbs*. Complex predicates involve a combination of a simplex verb, termed the *light verb*, and a noun, adjective, or prepositional phrase, termed the *non-verbal element*, or *NVE* (Dabir-Moghaddam 1995; Karimi 1997; Folli et al. 2005; Karimi-Doostan 2011). Common light verbs in Persian include *kardan* ‘to do/make,’ *shodan* ‘to become,’ *zadan* ‘to hit,’ *xordan* ‘to collide,’ *dâdan* ‘to give,’ and *dâshtan* ‘to have,’ among others. The examples below provide examples of complex predicates with a variety of light verbs and NVE types.

(15)

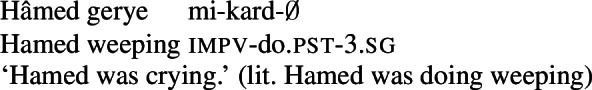

(16)

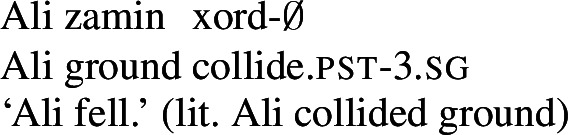

(17)

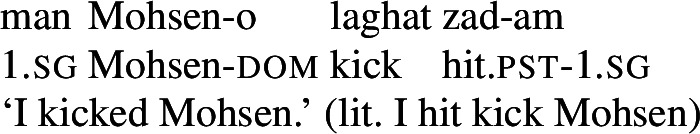

(18)

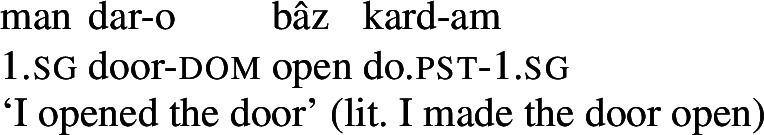

(19)
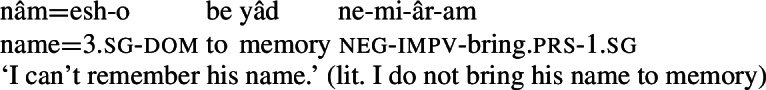

Complex predicates may be compositional, in which case the meaning of the entire complex predicate is predictable from the meanings of the light verb and NVE (Karimi 1997; Folli et al. 2005; Karimi-Doostan 2011). They can also be idiomatic, in which case the meaning of the complex predicate does not clearly follow from the meaning of its components. The distinction between compositional and idiomatic complex predicates has grammatical consequences. Importantly for the purposes of this paper, the NVE of compositional complex predicates can be modified using the *ezâfe* construction, as in (20) and (21).

(20)

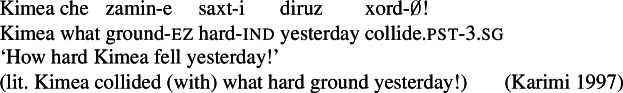

(21)
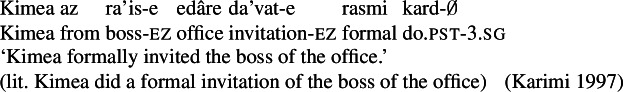

Idiomatic complex predicates, on the other hand, do not permit modification of their NVE. For example, the complex predicate *dust dâshtan* ‘to like, love’ (lit. have friend) does not permit modification of its NVE.

(22)
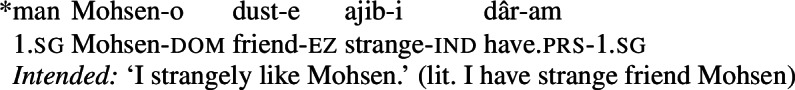

## Mental state predicates via possessive complex predicates

The complex predicate strategy extends as well to verbs expressing mental states. Specifically, a number of expressions that act as translational equivalents of English mental state verbs occur with the verb *dâshtan* ‘to have.’ (23)provides a non-exhaustive list of such possessive experiencer complex predicates.

(23)
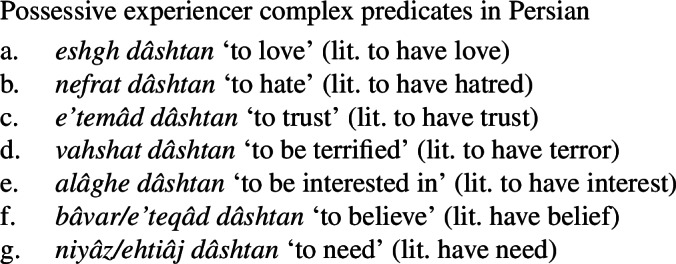

These complex predicates occur with an NP possessor acting as the experiencer of the mental state. The individual the experiencer bears the mental state toward, which I refer to as the target of the mental state, is introduced by a prepositional phrase, headed either by *be* ‘to’ or *az* ‘from, of,’ depending on the particular mental state nominal acting as the NVE.

(24)

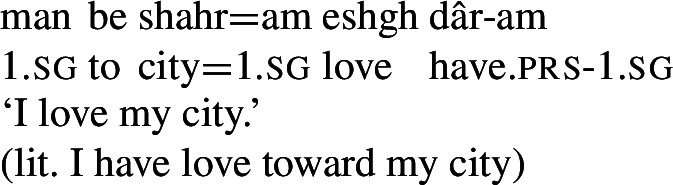

(25)

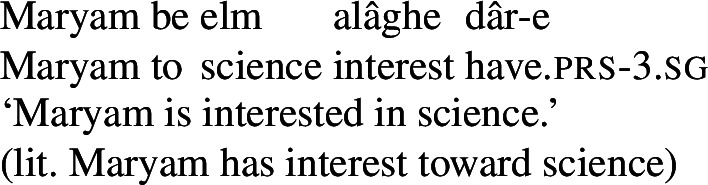

(26)

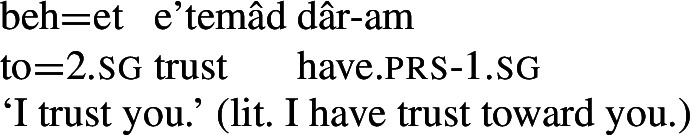

(27)

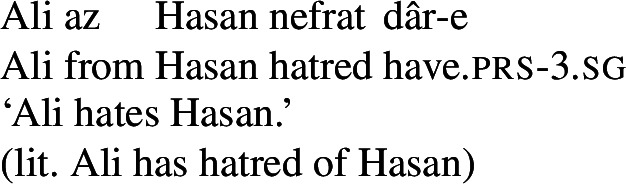

(28)

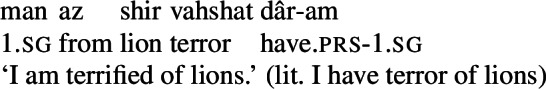

(29)

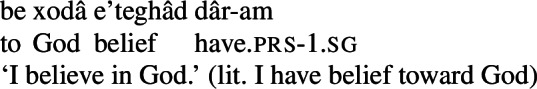

(30)
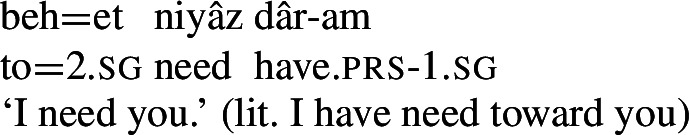

Possessive experiencer complex predicates are compositional rather than idiomatic in Karimi’s (1997) sense. This can be seen from the fact that the NVE can be modified by a range of adjectives by means of the *ezâfe* construction. Adjectives that may modify the NVE include *ajib* ‘strange,’ *shadid* ‘intense,’ and *kâmel* ‘complete,’ among others.

(31)

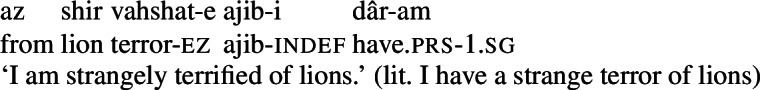

(32)

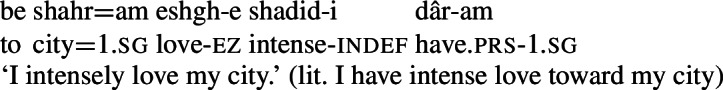

(33)

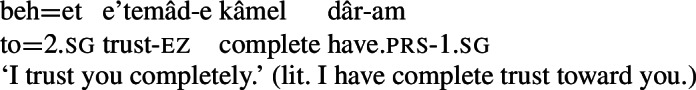

(34)
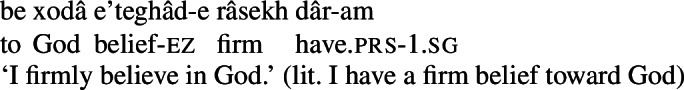

Moreover, the NVE of these complex predicates is gradable: the complex predicates in (23) above can be modified by *ziyâd* ‘much,’ intuitively referring to the “size” of the mental state.

(35)

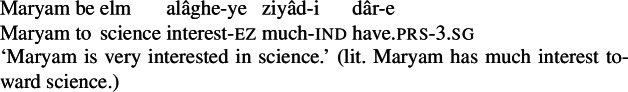

(36)
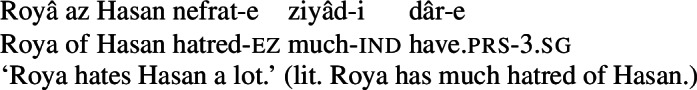

It is also possible to express comparison with possessive experiencer complex predicates by directly modifying the non-verbal element with the comparative *bishtar* ‘more.’ The expression of comparison in this way is completely productive: all of the complex predicates in (23) can be directly modified by *bishtar*. The standard of comparison is introduced by *tâ*, literally ‘until,’ which is used more generally with clausal comparison.^4^

(37)

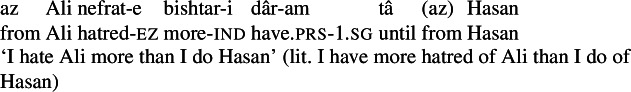

(38)

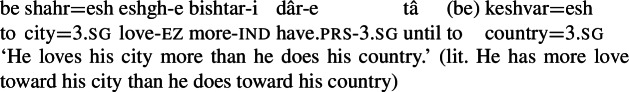

(39)

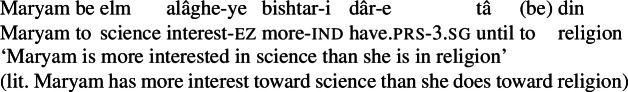

(40)
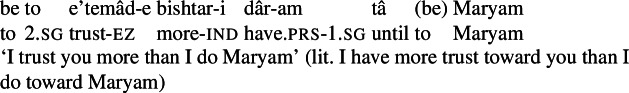

An analysis of possessive experiencer complex predicates should explain the ability of the non-verbal element to be modified by adjectives as an independent nominal, and should also account for its gradability. In addition to this, an ideal account would tie the analysis into a broader class of phenomena cross-linguistically.

## Possessive experiencer complex predicates as possessed property concepts

In what follows, I introduce Francez and Koontz-Garboden’s (2015, 2016, 2017) approach to property concept sentences as possession of portions of abstract qualities (see also Koontz-Garboden and Francez 2010; Hanink et al. 2019; Hanink and Koontz-Garboden 2021). I begin by discussing Francez and Koontz-Garboden’s results, motivate applying their general approach to possessive experiencer complex predicates by showing that the NVEs of such complex predicates pass Francez and Koontz-Garboden’s diagnostics for quality nouns, and then develop an initial analysis of possessive experiencer complex predicates using their framework. I ultimately show that applying their analysis with minimal modifications leads to problems in explaining certain properties of the Persian data, and motivate a revised analysis in Sect. 5.

### Possessive predicating strategies in property concept sentences

Francez and Koontz-Garboden observe that, cross-linguistically, property concept sentences, translational equivalents of what in English are expressed as predicative adjectival sentences, are often expressed via possession of an abstract mass noun, what they refer to as a *possessive predicating* strategy. Different languages may make use of a variety of possessive predicating strategies, including a nominal possessive strategy (Ulwa, (41)), a prepositional strategy (Hausa, (42)), or a verbal strategy (Wolof, (43)), among others. Crucially, the strategy used to express property concepts in these languages is the same strategy the language uses for possession more generally, as can be seen in the (b) sentences below.

(41)

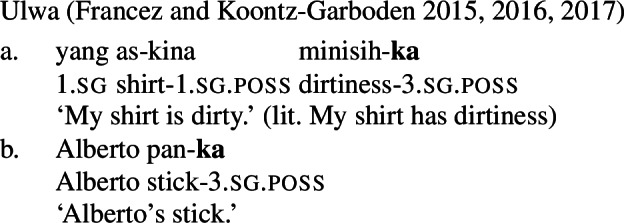

(42)

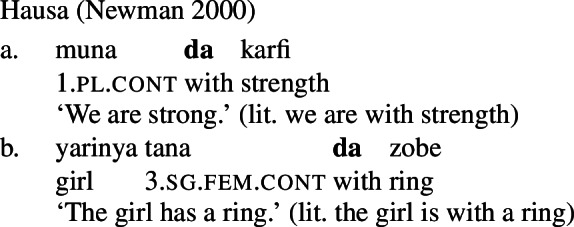

(43)
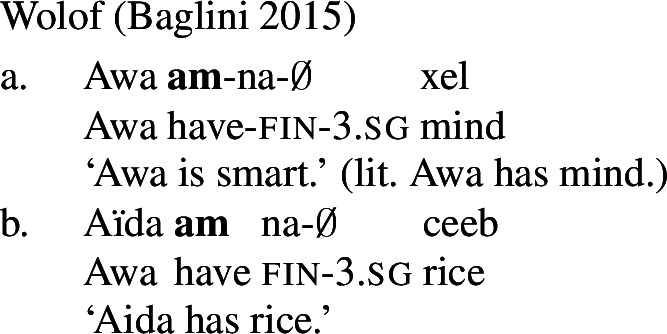

Francez and Koontz-Garboden develop an account of this phenomenon that takes the surface morphosyntax seriously. On their approach, the abstract noun denotes a set of portions of abstract qualities, themselves a subsort of individual.^5^ These qualities are mass entities ordered by two relations: a partial order interpreted as the parthood relation (Link 1983), and a total preorder that intuitively expresses the *size* of the portion of the quality in question. Finally, the possessive morphosyntax serves to existentially close the quality variable and relate it to an individual (the possessor) via the possessive relation *π*. The existential quantifier is restricted to those portions that “stand out” in the sense of Kennedy (2007), by virtue of having a significant or noteworthy size, or having a size few other portions have in the context. This is needed to model the fact that possessive predicating property concept sentences, much like their adjectival counterparts in other languages, are context dependent: (43-a) is true if Awa’s intelligence is sufficiently high to count as smart in the context. In the absence of any contextual restriction, (43-a) would be predicted to be true in contexts where Awa possesses any amount of intelligence, no matter how small. Such weak truth conditions, however, do not adequately capture the meaning of sentences like (43-a).

The context dependence of possessive predicating property concept sentences is implemented in Francez and Koontz-Garboden’s analysis by endowing the possessive verb with an *interval* argument, which is provided by the context. Here, an interval is a proper subset of portions in the denotation of the quality noun that exceed a threshold in the size preorder on qualities. More precisely, the interval is required to be *left-bounded*, in the sense that for some portion q in the denotation of the quality noun of which the interval is a subset, every portion in I exceeds q in the preorder. A formal definition from Francez and Koontz-Garboden (2017) is provided in (44).

(44) Interval: For any quality P, an interval I ⊂ P is a set of portions such that ∃q ∈ P[I = {p : q ≤ p}] (Francez and Koontz-Garboden 2017, p. 45, ex. (13))

We are now in a position to see how Francez and Koontz-Garboden’s analysis works. The Wolof sentence in (43-a) is analyzed as in (45): *xel* ‘mind’ is a predicate of portions of the quality mind. *am* ‘have’ existentially closes the property variable z and relates it to the individual *awa* via *π*. The notation ∃z^*I*^ expresses a restriction of the existential quantifier to portions in the interval I.^6^ The interval argument is further required to be a subset of the quality contributed by the abstract noun, here mind as contributed by *xel*.

(45)
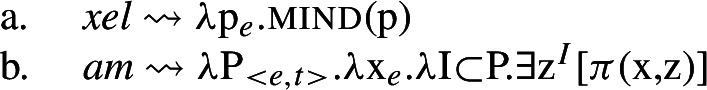

Francez and Koontz-Garboden (2017) follow Jacobson (1999) in adopting what they call a “directly compositional” approach to context dependence, on which context dependent expressions do not denote a proposition upon combining with all of their arguments. That is, just as for Jacobson a sentence containing a pronoun, such as *Mary likes him*, is analyzed as a function *λx.likes(Mary,x)* from individuals to truth values, Francez and Koontz-Garboden propose that the denotation of a possessive predicating property concept sentence is a function from intervals to truth values, as in (46-a). In order to have a truth value, context must provide an interval to saturate the interval argument of (46-a), delivering the truth conditions in (46-b).^7^

(46)
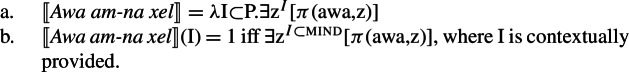

This analysis not only provides a compositional analysis of possessive predicating property concept sentences, but also accounts for their context dependence: because the contextually provided interval is a proper subset of the set of portions encoded by the quality noun, not every portion in the quality noun’s denotation will be high enough in the preorder to be an element of the interval. In this way, in the example above, Awa’s intelligence is required to stand out by virtue of being large enough to be included in the contextually provided interval of mind.

One can immediately appreciate the similarities between the phenomena Francez and Koontz-Garboden investigated and the Persian phenomena discussed in Sect. 3: these also involve a possessive predicating strategy for expressing a state. Crucially, *dâshtan* is used for possession more generally in Persian.

(47)

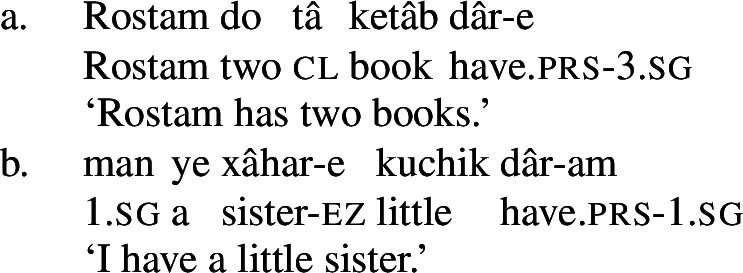

### Mental state nouns are quality nouns

The connection between property concept sentences and possessive experiencer complex predicates is deeper than a shared syntax: mental state nominals pass a range of diagnostics proposed by Francez and Koontz-Garboden (2017) to distinguish quality nouns from ordinary count and mass nouns. In what follows, I lay out Francez and Koontz-Garboden’s diagnostics for quality nouns, and then demonstrate that mental state nouns in English pass these diagnostics. I then show that Persian possesses analogous diagnostics that group mental state nouns with other quality nouns in the language, while distinguishing them from ordinary mass and count nouns.

First, quality nouns in English are known to permit *amount* readings with wh-exclamatives (48). This distinguishes them from concrete nouns, whether count or mass, which lack amount readings with wh-exclamatives (49) (Francez and Koontz-Garboden 2017).

(48)

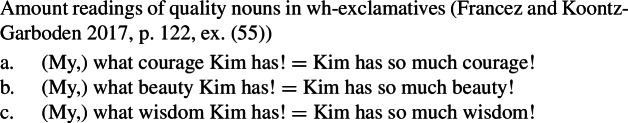

(49)
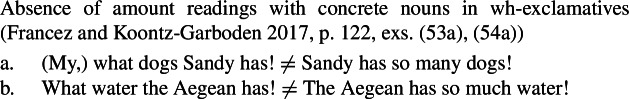

Wh-exclamatives with mental state nouns also possess amount readings in English, as (50) demonstrates.

(50)
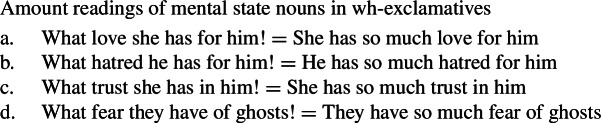

Second, using naturally occurring examples from the Web, and drawing on work by Morzycki (2012), Francez and Koontz-Garboden point out that quality nouns may be modified by modifiers in the *big* class, such as *big*, *huge*, and *major*, on a reading that measures the extent of a property (51), as well as by modifiers in the *utter* class, such as *utter*, *outright*, and *absolute* (52). Crucially, such modifiers are unacceptable with ordinary mass nouns (53). The idea is that quality nouns, though behaving like mass nouns in many respects, are inherently totally preordered by a size relation, while mass nouns are not, explaining the two classes’ differential modifiability by *big* and *utter* class modifiers.

(51)

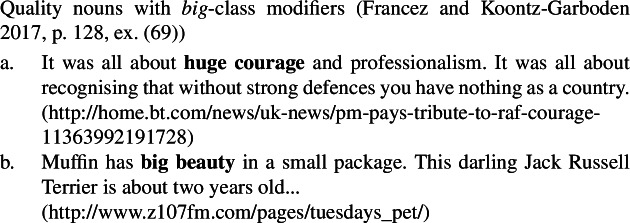

(52)

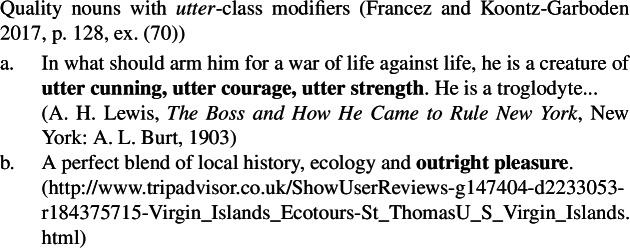

(53)
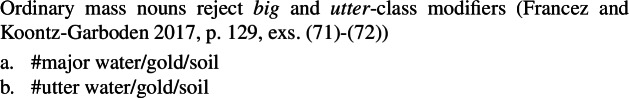

As can be seen in the natural occurring examples below, mental state nouns behave like other quality nouns in being compatible with both *big* class modifiers (54)and *utter* class modifiers (55), providing further support for the quality analysis of such nouns generally.

(54)

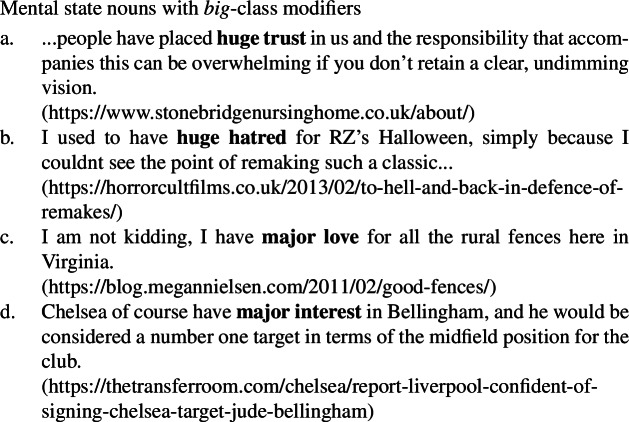

(55)
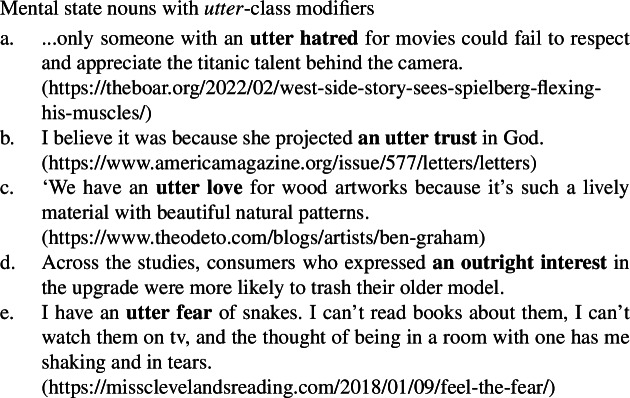

Furthermore, although not included in Francez and Koontz-Garboden’s set of diagnostics, mental state nouns (56)and quality nouns (57)pattern together in permitting modification by *utmost*, while ordinary nouns cannot be modified by *utmost* (58).

(56)
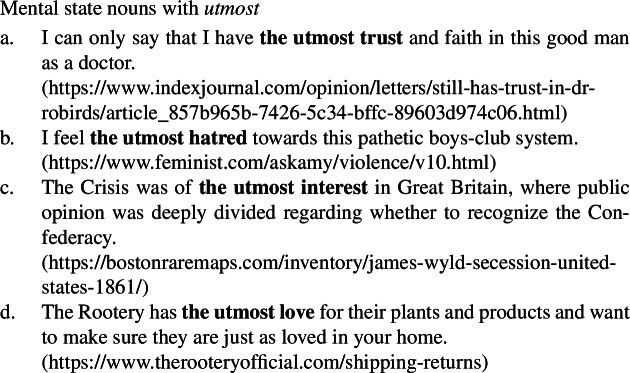

(57)

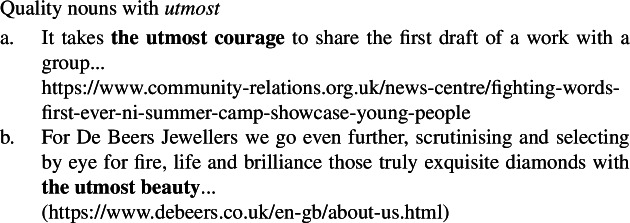

(58)
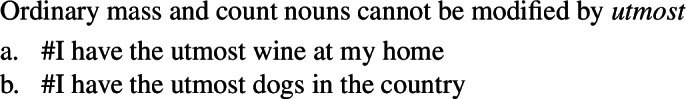

These diagnostics have analogues in Persian: just like their English mental state noun counterparts, the NVE of a possessive experiencer complex predicate behaves like a quality noun. First, the NVE can be used with a *che* ‘what’ exclamative, with the same amount reading as other quality nouns (59). Crucially, other quality nouns that do not refer to mental states, such as *zur* ‘strength’ and *jor’at* ‘courage,’ also possess amount readings with *che* exclamatives (60), while concrete count and mass nouns lack such a reading (61).

(59)

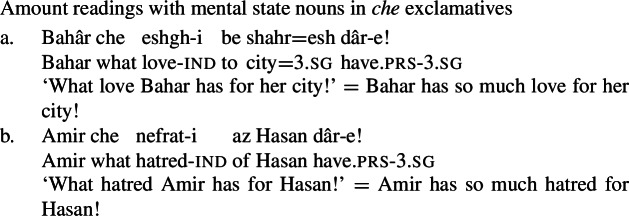

(60)

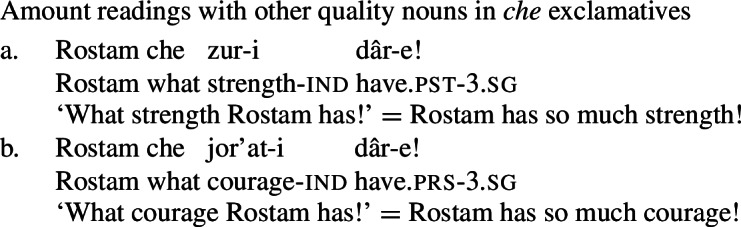

(61)
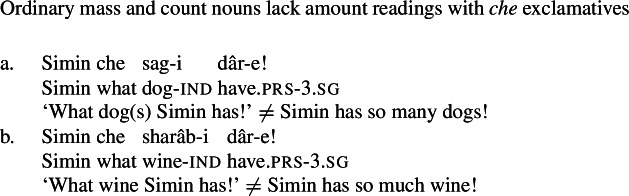

What’s more, Persian has an expression *nehâyat* ‘extremity, utmost’ which can compose with mental state nouns (62) as well as other quality nouns (63), but not with concrete nouns (64). This is reminiscent of the interaction between *big/utter*-class modifiers and quality nouns in English discussed above, and, more directly, the interaction of such nouns with *utmost*.

(62)
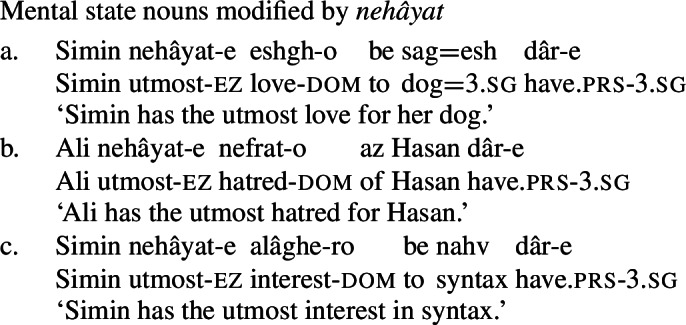

(63)

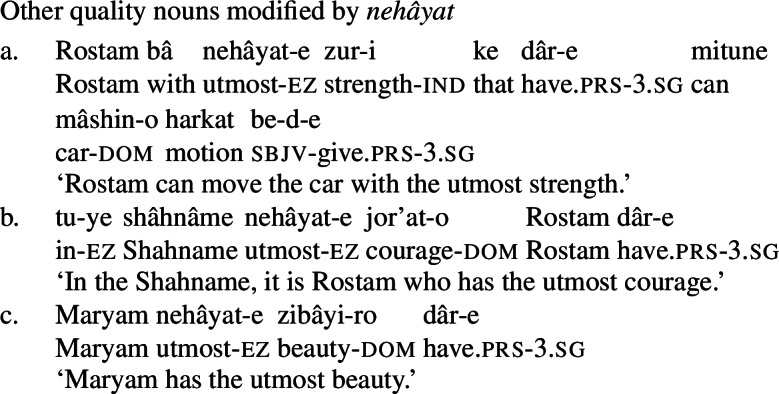

(64)
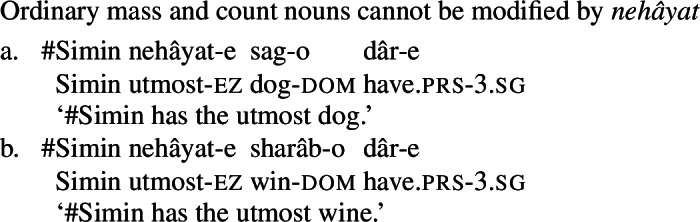

Altogether, these diagnostics strongly suggest that a quality semantics for mental state nouns in general, and for the NVEs of possessive experiencer complex predicates in particular, is on the right track.^8^

### Applying Francez and Koontz-Garboden’s analysis to possessive experiencer complex predicates

I propose that the possessed property concept analysis be extended to Persian possessive experiencer complex predicates. I begin by considering a direct application of Francez and Koontz-Garboden’s (2017) proposal to the Persian data, and ultimately argue for a modification of their approach to accommodate components of possessive experiencer complex predicates that are not shared with other possessive predicating property concept sentences.

On my proposal, as in Francez and Koontz-Garboden’s, the non-verbal element is a quality noun, and denotes a set of portions of an abstract quality corresponding to the mental state experienced. (65) demonstrates this analysis for *alâghe* ‘interest,’ which is treated as a predicate of portions of the quality interest.

(65) *alâghe* ⇝ *λ*p_*e*_.interest(p)

As for *dâshtan*, I follow Francez and Koontz-Garboden for the time being in analyzing it as in (45-b), where it introduces the possessive relation between a portion of a quality and an individual, existentially quantifies over the portion argument of the property concept, and requires that portion stand out with respect to the left-bounded interval I, as in (66).

(66) *dâshtan* ⇝ *λ*P_<e,t>_.*λ*x_*e*_.*λ*I⊂P.∃z^*I*^[*π*(x,z)]

The quality noun and the possessive verb compose straightforwardly by Function Application, yielding (67).

(67) *λ*x_*e*_.*λ*I⊂interest.∃z^*I*^[*π*(x,z)]

Before we can proceed to the truth conditions for sentences with possessive experiencer complex predicates, we need to consider the question of the semantics of target prepositional phrases and how they compose with the denotation of the complex predicate derived in (67). I turn to this in the next subsection, and show that it leads to problems for Francez and Koontz-Garboden’s approach.

### Problem 1: composing the target phrase

Possessive experiencer complex predicates differ from the property concept lexemes that Francez and Koontz-Garboden studied, by virtue of having not just a possessor, but also a PP argument expressing the *target* of the emotional state encoded in the NVE, the individual loved, hated, of interest, etc. These targets are introduced by the prepositions *be* ‘to’ or *az* ‘of/from,’ as discussed previously. This leads to our first compositional issue in applying Francez and Koontz-Garboden’s analysis to possessive experiencer complex predicates: there is no clear way to compose a possessive VP with a target phrase. The reason for this is that the possessive verb, upon composing with the quality noun, expects an individual argument representing the possessor of the quality, and an interval argument. While an interval on Francez and Koontz-Garboden’s approach is a set of portions itself, and the target phrase, if defined appropriately, could saturate this argument, this would interfere with the interval argument’s role in ensuring that the portion argument stands out in the context, and would cause further problems for the analysis of gradability on Francez and Koontz-Garboden’s analysis. I conclude that the target phrase cannot be identified with the interval argument, and thus that there is no way to compose the possessive VP with the target phrase on the analysis in Francez and Koontz-Garboden (2017).

The other possibility that would maintain Francez and Koontz-Garboden’s analysis of possessive VPs would be to treat target phrases as nominal modifiers, in which case they would compose with the quality noun rather than with the VP. An analysis that accomplishes this treats the prepositions *be* and *az* as functions from individuals to functions from predicates to predicates, where the individual argument stands in the Target relation to an individual (68).

(68) *be/az* ⇝ *λ*x_*e*_.*λ*P_<e,t>_*λ*y_*e*_.P(y) ∧ target(y) = x

Following composition with its first individual argument, the target phrase may compose with the quality noun, returning a predicate of portions which may in turn serve as the argument of *dâshtan*. Once the individual argument correspondsing to the possessor is saturated, we derive a predicate of left-bounded intervals of portions, completing the analysis. (69)provides the analysis of (25) on the approach developed thus far.

(69)

While this analysis works compositionally, it suffers from two problems. First, nominal modifiers, such as adjectival and prepositional phrases, *follow* the noun in Persian. This can be seen in the behavior of target phrases modifying quality nouns outside of possessive constructions, as in (70).

(70)
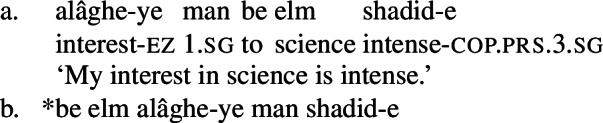

Prepositional phrases do precede bare noun objects in the VP, however, as can be seen in (71).

(71)
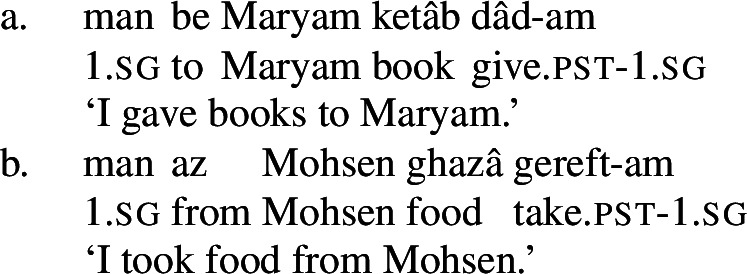

If target phrases modify the quality noun in a possessive experiencer complex predicate, it is strange that they would precede the noun. On the other hand, PP-Noun order is expected if the target phrase modifies the VP.

The second problem concerns the meaning of the sentence derived in (69). The predicted truth conditions for *Maryam be ’elm alâghe dâre* (lit. Maryam has interest toward science) are as in (72).

(72) 〚*Maryam be ’elm alâghe dâre*〛 = 1 iff ∃z$^{I\subset \{\text{p: \textsc{interest}(p)} \wedge\text{ \textsc{target}(p)} = \textit{science}\}}$[*π*(*maryam*,z)]

In words, the sentence is true iff Maryam has a portion in the contextually provided interval, which is required to be a subset of portions of interest whose target is science. While this does correctly require that the portions at issue have science as their target, the consequence is that Maryam’s portion of interest must stand out specifically with respect to portions of interest whose target is science, rather than portions of interest more generally. Intuitively, this seems to be too restrictive: a portion of interest should stand out with respect to other portions of interest, rather than just those with the same target. More seriously, this leads to a problem for the analysis of comparisons between portions with different targets, which I take up in the next section.

### Problem 2: gradability and comparison of VPs and quality nouns

On Francez and Koontz-Garboden’s approach, the gradability of possessive predicating property concept sentences is handled by appealing to the left-bounded interval argument of the possessive verb. The comparative, whose denotation is given in (73), takes the VP as an argument, along with the comparative standard and the main clause subject, and asserts that the set of intervals derived by applying the VP denotation to the comparative standard is a subset of the set of intervals derived by applying the VP to the main clause subject.

(73) *λα*.*λ*x_*e*_.*λ*y_*e*_.*α*(y) ⊂ *α*(x), where *α* is a function of type <e<<p,t>,t>>^9^

They then apply this approach to the analysis of comparatives in the Misumalpan language Ulwa, which makes use of possessive predicating property concept sentences in the form of quality nouns with a possessive suffix *-ka*. The comparative applies to the possessed NP, and, upon composing with the standard phrase and the subject, returns true iff the set of intervals of tallness containing Clementina’s portion of height is a subset of the set of intervals containing Abanel’s portion of height (74-b).

(74)
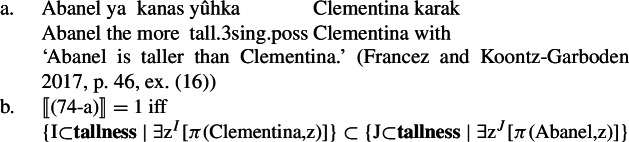

The denotation of the comparative in Francez and Koontz-Garboden is designed to handle possessive VP comparisons in Ulwa. If we adapt this approach to Persian, we expect that comparatives and other degree modifiers should be able to modify VPs. This turns out to be correct: both *ziyâd* ‘much’ and *bishtar* ‘more’ can adjoin to the VP, as the following examples show.

(75)
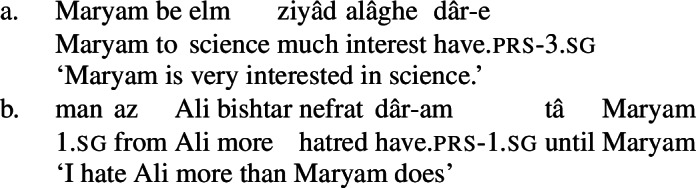

A straightforward application of Francez and Koontz-Garboden’s analysis predicts the availability of VP comparisons. However, there are two major issues with their approach. First, while the analysis in (73) is able to handle possessor comparisons like that in (75-b), where the target and standard of comparison involve distinct possessors, it does not extend to comparisons involving distinct targets, as in (76).

(76)
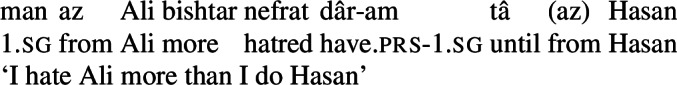

To see this, recall that, on the assumption that target phrases compose with the quality noun, the interval argument of the possessive VP is restricted to sets of portions of a certain kind with a specific target. In the case of (76), for instance, we predict the meaning of the main clause VP to be as in (77-a), where the interval argument is restricted to sets of portions of hatred whose target is Ali. This leads to the truth conditions in (77-b).

(77)
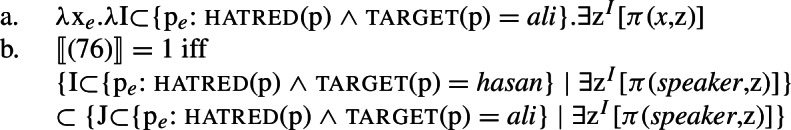

On the reasonable assumption that portions have unique targets,^10^ the set of intervals of portions of hatred whose target is Hasan is disjoint from the set of intervals of portions of hatred whose target is Ali. As it is not possible for the former set to be a subset of the latter, we make the undesirable prediction that comparitives like (76) are contradictions.^11^

The second problem concerns the ability of *ziyâd* and *bishtar* to directly modify the quality noun, as discussed in examples (35)-(40) in Sect. 3, some of which I repeat below for convenience. That *ziyâd* and *bishtar* are indeed modifying the noun can be seen by the presence of *ezâfe*, which, as noted above, appears between the noun and any adnominal modifiers.

(78)
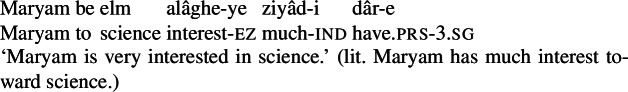

(79)
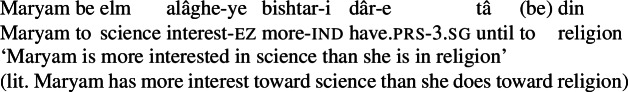

On Francez and Koontz-Garboden’s analysis, the comparative takes arguments of type <e<<p,t>,t>>. As such, it simply cannot compose with the quality noun, which is of type <p,t>. We thus have no analysis of the gradability of the quality noun independently of the possessive VP in which it is contained.^12^

### Interim summary

I have argued here that a direct application of Francez and Koontz-Garboden’s analysis with minimal modifications to possessive experiencer complex predicates leads to problems with the analysis of target phrases and gradability. In what follows, I propose modifications to Francez and Koontz-Garboden’s analysis that overcomes the problems discussed above, in addition to making a variety of additional desirable predictions.

## A new possessive predicating analysis for possessive experiencer complex predicates

In this section, I propose a possessive predicating analysis of possessive experiencer complex predicates that builds off of Francez and Koontz-Garboden’s work, but makes modifications to their analysis in order to solve the problems noted in the previous section.

First, I continue to maintain Francez and Koontz-Garboden’s analysis of quality nouns as predicates of portions; the analysis of a quality noun will thus continue to be as in (65) above, repeated as (80) below for convenience.

(80) *alâghe* ⇝ *λ*p_*e*_.interest(p)

I also maintain my proposal for the analysis of target phrases as functions from predicates to predicates, with target prepositions being functions from individuals to such functions, as in (68). The analysis of the target phrase *be/az Ali* will thus be as in (81).^13^

(81) *be/az Ali* ⇝ *λ*P_<e,t>_.*λ*p_*e*_.P(p) ∧ target(p) = *ali*

### The meaning of the possessive verb

The first modification I make to Francez and Koontz-Garboden’s analysis concerns the denotation of the possessive verb *dâshtan*. I arrive at the new analysis by removing the interval argument and the existential quantification over the portion argument and lambda abstracting over the portion argument. The revised analysis of *dâshtan* is thus as in (82).

(82) *dâshtan* ⇝ *λ*P_<e,t>_.*λ*x_*e*_.*λ*p_*e*_.P(p) ∧ *π*(x,p)

As in Francez and Koontz-Garboden’s approach, *dâshtan* takes the quality noun as an argument. Upon doing so, the result is a function from individuals to predicates of portions, as in (83).

(83) *λ*x_*e*_.*λ*p_*e*_.interest(p) ∧ *π*(x,p)

Because the portion argument is available for composition at the VP-level, we can compose the target phrase with the possessive VP in a way that overcomes the previously noted problems with Francez and Koontz-Garboden’s analysis. I propose that the target phrase, analyzed as a function from predicates to predicates, composes with the possessive VP via a standardly available rule of function composition, defined in (84).

(84) Function Composition
  f ∘ g = *λ*y.f(g(y))

Essentially, Function Composition works by saturating an argument of one function, applying the second function to the result, and then abstracting over the saturated argument place of the first function. This allows us to compose the target phrase with the possessive VP despite the presence of the possessor argument. The compositional process is shown for our running example, repeated in (85), in (86).^14^

(85)
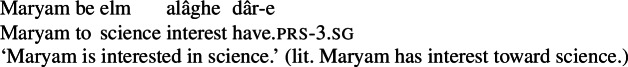

(86) 〚be ’elm alâghe dâre〛
  = 〚be elm〛 ∘ 〚alâghe dâre〛
  = *λ*y_*e*_.〚be elm〛(〚alâghe dâre〛(y))
  = *λ*y_*e*_.(*λ*P_<e,t>_.*λ*p_*e*_.P(p) ∧ target(p) = *science* *λ*x_*e*_.*λ*p_*e*_.interest(p) ∧ *π*(x,p) (y))
  = *λ*y_*e*_.(*λ*P_<e,t>_.*λ*p_*e*_.P(p) ∧ target(p) = *science* *λ*p_*e*_.interest(p) ∧ *π*(y,p))
  = *λ*y_*e*_.*λ*p_*e*_.interest(p) ∧ *π*(y,p) ∧ target(p) = *science*

Once the target phrase composes with the possessive VP, all that is left is to saturate the possessor argument by Function Application. Once this is done, we have a nearly complete analysis of basic examples like (85), the analysis of which is as in (87).

(87) *λ*p_*e*_.interest(p) ∧ *π*(*maryam*,p) ∧ target(p) = *science*

Finally, we need to saturate the portion argument, so that a sentence with a possessive experiencer complex predicate is truth-evaluable. I propose that this be accomplished by a rule of existential closure, defined in (88).

(88) ∃(P) → ∃p_*e*_[*C*(p) ∧ P(p)]

Here, the existential quantifier encodes an additional restriction on portions, *C*, which can be construed as the property of standing out in the context, in much the same way as Francez and Koontz-Garboden’s left-bounded interval argument. In other words, the existential quantifier requires not only that a portion with the property P exist, but also that it stand out in the context.^15^ The tree in (89)provides an illustration of the complete compositional analysis of our running example in (85).

(89)

### Gradability and comparison

The second major modification I make to Francez and Koontz-Garboden’s analysis involves the approach to gradability. Having removed the interval argument from the possessive VP, I cannot make use of Francez and Koontz-Garboden’s analysis of the comparative.

Instead, I adopt an analysis of the comparative based on the use of *measure functions*, following Wellwood (2014). On this approach, the comparative introduces an underspecified measure function *μ*, which directly measures the size of the portion argument and compares it to the maximal degree of the measure in the standard clause headed by *tâ*.^16^

(90) *bishtar* ⇝ *λ*d_*d*_.*λ*P_<e,t>_.*λ*p_*e*_.P(p) ∧ *μ*(p) > d

Upon composing with the degree denoted by the standard clause, *bishtar* may compose with the quality noun directly via Function Application. The compositional analysis of a nominal comparative like (39), repeated in (91), is provided in (92), where *δ* represents the degree contributed by the standard phrase headed by *tâ*.^17^ (93) provides the full truth conditions for the comparative.

(91)
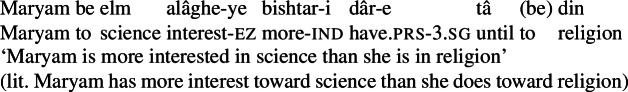

(92)

(93) ∃p_*e*_[*C*(p) ∧ interest(p) ∧ *π*(maryam,p) ∧ target(p) = science ∧ *μ*(p) > max(*λ*d_*d*_.∃p’_*e*_[interest(p’) ∧ *C*(p) ∧ *π*(maryam,p’) ∧ target(p’) = religion ∧ *μ*(p’) ≥ d])]

On this analysis, a quality noun comparative like (91) is true if Maryam’s portion of interest toward science has a greater measure than that of her portion of interest toward religion, as desired.

What’s more, the analysis already has the means to analyze VP comparisons: just as target phrases may compose with the possessive VP via Function Composition, *bishtar* may compose with the VP in the same fashion. This means that we can analyze sentences like (76) or the example in (94), chosen to be parallel to (91) analyzed in (93).

(94)
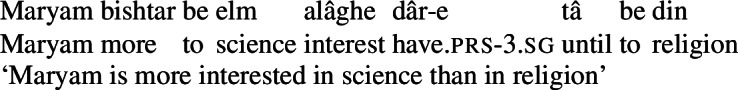

The tree below provides the compositional analysis of (94).

(95)

The meaning derived in (95)is the same as that derived for quality noun comparison as in (93), as desired. We are thus able to analyze both VP and quality noun comparisons in Persian.

To conclude this section, I provide an analysis of *ziyâd* ‘much.’ I posit a similar entry to that for *bishtar*, but have the measure of the quality greatly exceed the contextual standard of the measure function (96).^18^

(96) *ziyâd* ⇝ *λ*P_<e,t>_.*λ*p_*e*_.P(p) ∧ *μ*(p) >> std(*μ*)

(98)gives the analysis of (36), repeated in (97), with *ziyâd* composing with the NVE once again by Function Application. As with *bishtar*, *ziyâd* may compose with both the quality noun and the possessive VP, the latter via Function Composition, with the same meaning in (98), as desired.

(97)
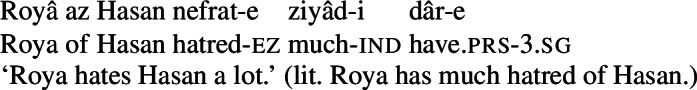

(98) ∃p_*e*_[*C*(p) ∧ hate(p) ∧ *μ*(x) >> std(*μ*) ∧ *π*(roya,p) ∧ target(p) = hasan]

Here, the gradability of the NVE is guaranteed without requiring that quality nouns have the same type as possessive VPs, as required for Francez and Koontz-Garboden’s analysis of comparatives in Ulwa, nor does it require recourse to another strategy treating quality nouns as denoting measure functions or being otherwise endowed with a degree argument (Morzycki 2009). Rather, reference to degrees is contributed by functional material, with the noun contributing a property of measurable individuals.

### Addressing the problems from Sect. 4

The analysis developed in the previous subsections addresses all of the problems that arise when applying Francez and Koontz-Garboden’s analysis with minimal modifications, discussed in Sect. 4. First, it solves the problem of the composition of the possessive VP with target phrases. Second, precisely because target phrases can compose with the possessive VP, rather than the quality noun, their position to the left of the noun is unsurprising: their position is simply the expected position of VP modifiers. Third, the new analysis does not predict that comparatives with distinct target phrases are contradictions, but instead captures their correct truth conditions. Finally, my analysis accommodates the gradability of the possessive VP in Persian as well as the gradability of the quality noun itself, and thus improves on Francez and Koontz-Garboden’s analysis, which accommodates the former but not the latter.

In addition to presenting a solution to the problems noted in Sect. 4, the analysis makes a number of additional predictions that I show to be correct below.

### Additional predictions

First of all, if the non-verbal element denotes a predicate of portions of a quality, and such portions are in turn a subsort of individual, their ability to be modified by adjectives, as demonstrated in examples (31)-(34), is expected. For example, if an adjective phrase like *ajib* ‘strange’ is treated as a predicate modifier, then it can compose with a predicate of portions straightforwardly, as in (99).

(99)
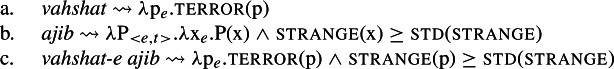

Second, since target phrases are treated as functions from predicates to predicates on my analysis, we expect that they should be able to compose not only with the possessive VP by function composition, but also directly with the NVE via Function Application, and that they should be able to appear with the NVE in its nominal use outside of complex predicates. This prediction is borne out: the target phrase may appear with independent mental state nominals. Further, as noted previously, the target phrase occurs to the *right* of the quality noun, as expected for nominal modifiers in Persian more generally.

(100)
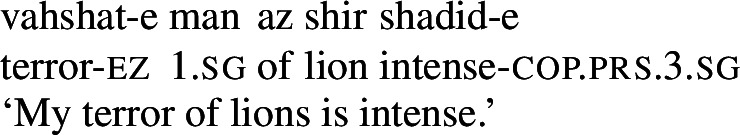

(101)
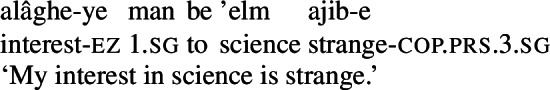

Note that these examples are definite. This is easily accommodated on my analysis, given the type of quality nouns as properties and the independently motivated presence of Partee’s (1987) *ι* type-shifter in Persian (Jasbi 2020). They can be analyzed as in (102), with *ι* applied to the subject.

(102) intense(*ι*p_*e*_[terror(p) ∧ target(p) = ^∩^lion ∧ *π*(*speaker*,p)])

A final prediction concerns the behavior of comparatives with possessive experiencer complex predicates. Recall that on my analysis existential closure applies to saturate the portion variable, delivering the truth conditions in (93), repeated in (103)for convenience.

(103) ∃p_*e*_[*C*(p) ∧ interest(p) ∧ target(p) = science ∧ *π*(maryam,p) ∧ *μ*(p) > max(*λ*d_*d*_.∃p’_*e*_[*C*(p) ∧ interest(p’) ∧ target(p’) = religion ∧ *π*(maryam,p’) ∧ *μ*(p’) ≥ d])]

Note that the existential quantifier comes with a requirement that the portion it quantifies over stand out in the context. This amounts to a prediction that comparatives with possessive experiencer complex predicates entail the positive: in other words, we expect (37), repeated in (104), to entail hatred of Ali and Hasan, and (39), repeated in (105), to entail interest in science and religion.

(104)
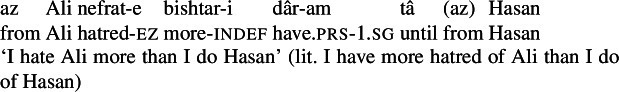

(105)
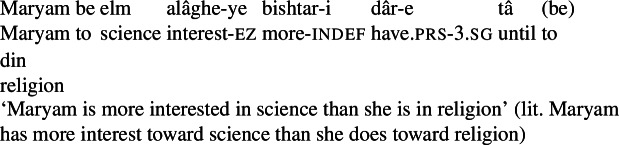

This prediction turns out to be correct: comparatives with possessive experiencer complex predicates entail reaching a positive threshold for the possessed mental state. In particular, (106)and (107)demonstrate that it is not possible to deny hatred/interest for the target if a comparative has been uttered in the previous discourse.^19^

(106)
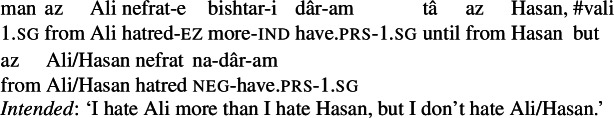

(107)

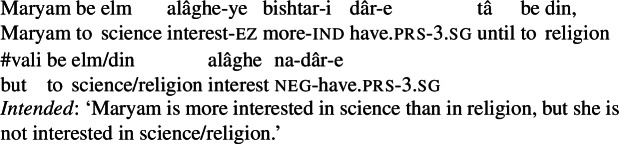

### Apparently recalcitrant data: relativized and quantified NVEs

To conclude this section, I turn to an apparent problem for the analysis. On my analysis, as on Francez and Koontz-Garboden’s, the first argument of the possessive verb is a predicate of individuals, corresponding to the quality noun, which, in all examples discussed thus far, denotes a set of portions of an abstract quality. However, there appear to be cases in Persian in which the NVE of a possessive experiencer complex predicate does not obviously denote a predicate. In particular, there are examples in which the NVE is relativized (108), questioned (109), or quantified (110).^20^

(108)
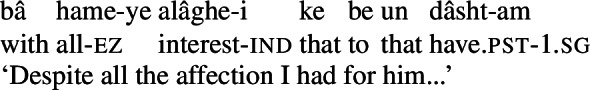

(109)

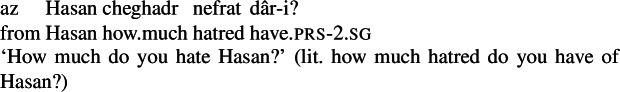

(110)
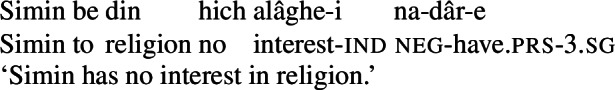

Regarding (108), on most approaches to the structure and interpretation of relative clauses (Sauerland 1998; Bhatt 2002; Hulsey and Sauerland 2006), the moved component, whether a null operator or the head noun, depending on the analysis, leaves a trace of type *e*. Since *dâshtan* expects a predicate of portions as an argument, the trace, being of type *e*, cannot compose with *dâshtan*, and a type clash results. Similarly, *wh*-phrases and quantified NVEs are scope-taking elements, and, on a Quantifier Raising analysis (May 1985; Kratzer and Heim 1998), would be structurally and semantically parallel to the relative clause case. We would therefore expect a type *e* trace as the argument of *dâshtan* in these examples, and, all else being equal, the analysis would (108)-(110) to be unacceptable, contrary to fact.

It turns out, however, that there is a solution to this apparent puzzle that is fully compatible with an analysis in which *dâshtan* takes a property argument. In particular, if the trace of movement is of type *e*, it can be shifted to a predicate via Partee’s (1987) ident type-shifter, defined below.^21^

(111) ident(c) := *λ*y_*e*_.y = c

The ident-shifted trace is now of type <e,t>, and is thus an appropriate argument for *dâshtan*. A derivation using this approach is shown in (113), based on the quality NP with a relative clause in (108), repeated below for convenience.

(112)
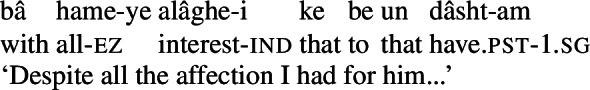

(113)

Similar analyses are possible for (109) and (110): both involve scope-taking elements that can be analyzed in terms of (covert) Quantifier Raising, leaving a trace that can be converted into a predicate via ident. The upshot is that the existence of moved and quantified NVEs does not present a technical problem for a possessive predicating analysis on which *dâshtan* takes a predicate argument.

## Beyond Persian: mental state nouns and possessive predication cross-linguistically

While I have focused on Persian in this paper, my analysis receives independent support outside of Persian. For one, possessive-predicating strategies for the expression of mental state is not limited to Persian, and is attested in several other languages in a way that tracks the morphosyntactic strategy used more broadly in the language in question. Moreover, several proposals have been made in syntactic theory that decompose stative transitive verbs into possessive structures (Noonan 1992; Hale and Keyser 1999, 2002), suggesting that the approach can be applied to languages like English as well.

### Possessive-predicating strategies for mental state expressions outside Persian

Persian is not alone in constructing mental state predicates by means of a possessive predicating strategy. For one, one can see from the examples in Sects. 1 and 4.2 that both English and Italian are able to make use of a possessive predicating strategy for the expression of mental states, even if this is not the most common strategy utilized by these languages. This said, there are a number of languages that use possessive predication as a primary strategy to express mental states. Moreover, we see the same kind of variation in possessive predication as Francez and Koontz-Garboden (2017) observed for other kinds of property concept sentences. For example, in Sorani Kurdish, a Northwestern Iranian language, mental state predicates exist that are expressed using an existential construction with a prepositional target phrase (114), where the possessor is expressed as a possessive clitic on the possessed NP, amounting to what Francez and Koontz-Garboden (2017) call a *existential possessive NP strategy* (114). In addition to the clitic, the possessor may be expressed as a topic phrase at the beginning of the sentence. Importantly, this existential construction is the same one used for possession in the language more generally (115).^22^

(114)
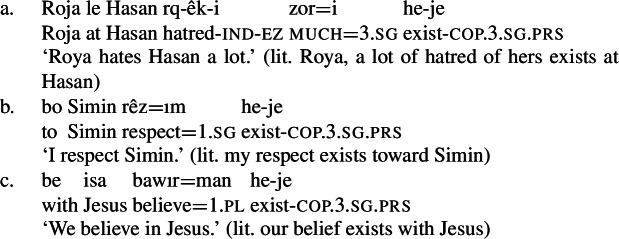

(115)
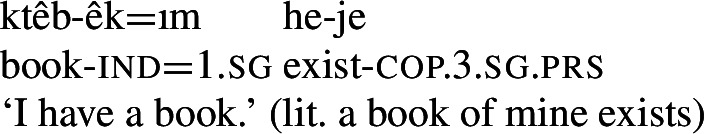

Even outside of the Iranian language family, one can find languages that express mental states by means of a possessive strategy. For instance, the Celtic languages Irish and Scottish Gaelic make use of an existential construction to express both possession and mental state predicates, using what Francez and Koontz-Garboden (2017) call a *property pivot strategy*, in which the quality noun is the existential subject, and both the possessor of the quality and its target are introduced by prepositional phrases. A closer look at Irish reveals that such a possessive predicating strategy exists for many of the same predicates that Persian constructs with *dâshtan*. The following examples are drawn from Noonan (1992) and from *Foras na Gaeilge*’s Irish dictionary.^23^

(116)
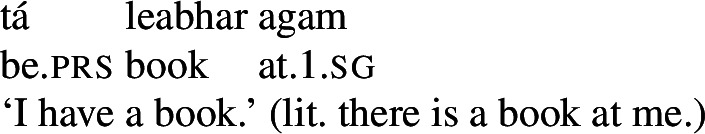

(117)

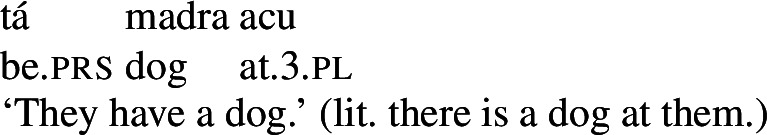

(118)

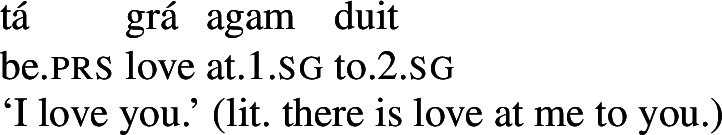

(119)

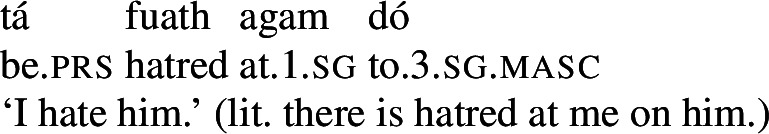

(120)

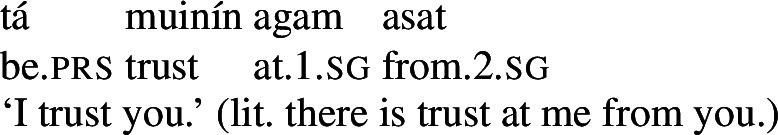

(121)

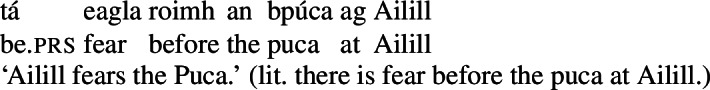

(122)
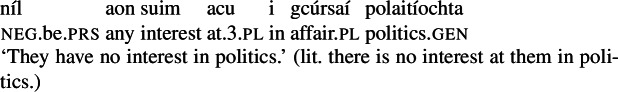

The same analysis I have proposed for Persian can be extended to Sorani and Irish, differing only in how the components of the meaning are distributed throughout the structure.^24^ For example, the existential possessive NP strategy of Sorani can be analyzed by having the possessive clitic introduce possessive semantics, with the prepositional phrase introducing the target (124).

(123)
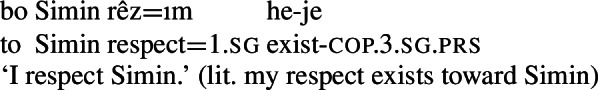

(124)
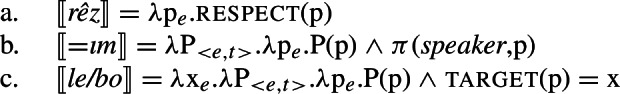

The clitic composes directly with the quality noun, while the target preposition composes with an individual, producing a phrase of type <p,t>, which can in turn compose with the quality NP. (125)provides a compositional analysis for (123)using the proposal in (124).^25^

(125)

The analysis of Irish is essentially analogous to that of Sorani immediately above. The difference would lie in that the possessor is introduced by the preposition *ag*, with a semantics as in (126).

(126) 〚*ag*〛 = *λ*x_*e*_.*λ*P_<e,t>_.*λ*p_*e*_.P(p) ∧ *π*(x,p)

This analysis predicts quality nouns to be modifiable and gradable like their Persian equivalents, and to otherwise behave like quality nouns in the language. The fact that they can be so modified can already be seen in examples above: for example, in the Sorani sentence in (114-a), *rqek* ‘hatred (indef.)’ is modified by *zor* ‘much’ using the *ezâfe* construction, in the same way that Persian NVEs can be modified by *ziyâd* ‘much.’ Likewise, in the Irish sentence in (122), *suim* ‘interest’ appears with a quantifier *aon* ‘any,’ suggesting these may permit quantification in a manner similar to Persian NVEs. I leave further exploration of the properties of these constructions to future research.

### Decomposing transitive stative verbs

A number of syntactic approaches to verbal argument structure have proposed that stative transitive verbs of the sort found in English be decomposed into an underlying mental state noun and a possessive element, either a preposition or a verb (Noonan 1992; Hale and Keyser 1999, 2002; Harves and Kayne 2012). For example, Noonan (1992) decomposes both English *love* and its Irish possessive equivalent into a possessive structure containing a stative NP and a possessive verb have (127).

(127) Decomposition of *love* from Noonan (1992: 201, ex.21)

In a similar vein, Hale and Keyser (2002) point out that English has several possessive paraphrases for stative transitives, alternating with a *have* and *give* form where the root of the verb is realized as a nominal.

(128)
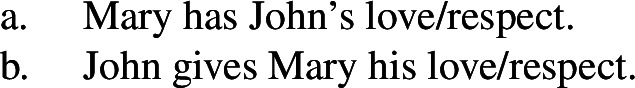

For this, and other reasons, such as the inability of stative transitives to form middles, Hale and Keyser decompose them into a combination of a verb and a prepositional phrase, with the target phrase in the specifier of the prepositional phrase and the mental state expressed as a noun acting as the complement of P.

(129) Hale and Keyser’s (2002, p. 41, ex. (26)) decompositional analysis of *respect the truth*

Given this existing tendency in the syntactic literature to decompose stative transitive verbs into possessive predicating structures, it is perhaps no surprise that there should be languages that express mental states primarily via possessive predication: languages like Persian simply express on the surface an underlying structure common to all languages. What’s more, this means that the analysis I have proposed for Persian is applicable to languages like English. As an example, the syntax in (129)could be endowed with a quality semantics similarly to what I proposed for Persian, with the P head taking the quality noun as its first argument, followed by the DP argument in its specifier.

(130)
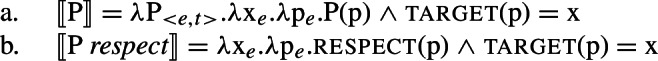

Even outside of decompositional approaches to the argument structure of stative verbs, there is reason to think that stative transitive verbs share a common, quality-based core with possessive predicating property concept sentences. For instance, Baglini (2015) and Francez and Koontz-Garboden (2017) point out that, in Wolof, possessive predicating property concept lexemes pattern with stative transitive verbs in, e.g., their compatibility with the degree modifier *lool* ‘very’ and the comparative *gën*. Non-stative predicates and possessive VPs with mass nouns are unacceptable with both, suggesting that quality possession is at issue.

(131)
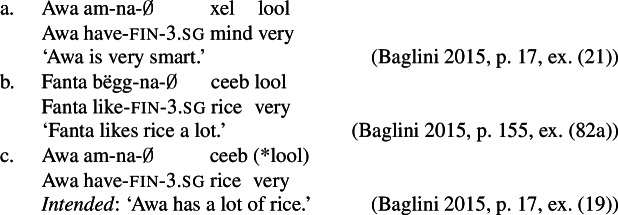

In this way, the semantics of quality possession can be extended straightforwardly to languages that express mental states via transitive verbs, in much the same way that such a semantics can be extended to lexical adjectives for property concept sentences more generally (Menon and Pancheva 2014).

## Taking stock and areas for future research

I have examined the properties of possessive experiencer complex predicates in Persian, demonstrating the modifiability and gradability of their NVEs. I proposed that they be analyzed as instances of possessive predicating property concept sentences, extending Francez and Koontz-Garboden’s semantics for quality possession to mental state constructions, while enriching the general approach with an analysis of target phrases as predicate modifiers, an existential closure rule, and a measure function approach to comparatives and *ziyâd* (Wellwood 2014). I showed that the approach captures key properties of these constructions, such as their modifiability and gradability, and makes further correct predictions about their behavior. I then showed that the proposal can be extended to explain the properties of expressions encoding mental state outside of Persian by showing that the possessive predicating strategies for mental states in languages like Sorani Kurdish and Irish can be endowed with a similar analysis to Persian and extending the approach to a decompositional analysis of mental state verbs in the style of Hale and Keyser (2002).

In addition to explaining the properties of possessive experiencer complex predicates in Persian, this work forges a more general connection between the grammar of mental state predicates and that of property concept sentences cross-linguistically. In particular, this work provides direct support for Baglini (2015) and Francez and Koontz-Garboden’s (2017) approach that posits a shared ontology between possessive predicating property concept sentences and transitive stative verbs, by virtue of the fact that the latter are instantiated by possessive predication in some languages. It further develops a semantics that aligns well with extant analyses in the syntactic literature that take stative transitives to be decomposed into a nominal head expressing a mental state and a prepositional or verbal head expressing possessive or target semantics.

This work leads to a number of directions for future research. In what follows, I discuss a few of these areas, as well as questions raised by the work that, though I provide preliminary answers to here, certainly require further investigation.

### Cross-linguistic variation

In Francez and Koontz-Garboden’s (2017) approach, qualities and the nouns that characterize them see very little compositional action, as they are existentially quantified off by the possessive very early in the derivation of property concept sentences. However, given the gradability of quality nouns in Persian, along with their greater degree of syntactic freedom, it would be illuminating to revisit Francez and Koontz-Garboden’s original domain of inquiry, and examine the gradability of the quality nouns themselves in languages with a possessive predicating strategy for property concept sentences more generally. For example, do languages like Wolof and Hausa show evidence of the gradability of the quality noun independently of the possessive verb phrase, and can they be moved or quantified? Or do quality nouns vary in their syntactic independence and gradability, such that gradability only arises in the possessive construction?

### Possessive attitudinal complex predicates

While in this paper I have concentrated on mental state predicates corresponding to certain kinds of emotional states, such as love and hatred, Persian also makes use of a possessive predicating strategy to express a number of propositional attitude predicates, particularly concerning belief, desire, and need. (132)gives several examples of such complex predicates.

(132)
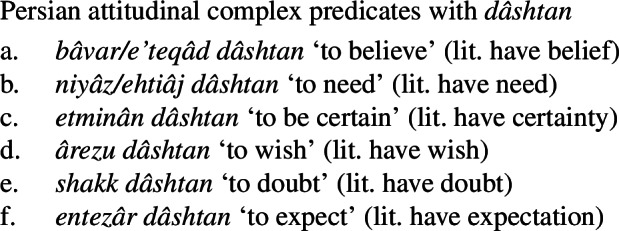

Like possessive experiencer complex predicates, these expressions may appear with a target phrase (133). Given their nature as propositional attitude predicates, they may also take clausal complements expressing the content of the attitude (134).

(133)
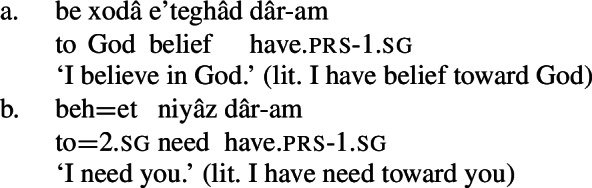

(134)
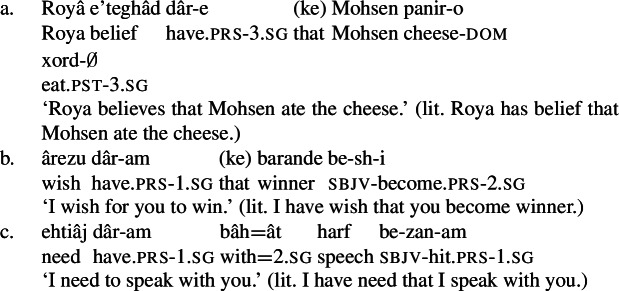

Also like possessive experiencer complex predicates, the NVE of these complex predicates is modifiable.

(135)
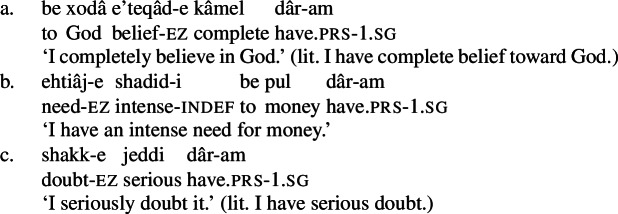

While I leave an in-depth study of this class of complex predicates to future research, a promising approach would be to treat the NVEs of these complex predicates as content nouns, building on analyses of clausal embedding (Kratzer 2006, Moulton 2009, Elliott 2017). This would involve embedding a possible worlds semantics for attitude predicates within the quality semantics, with possessive predication introducing the attitude holder. Such an approach would deliver a unified analysis for possessive predicating mental state predicates in Persian, while also accounting for the properties particular to attitudinal complex predicates.

### The typology of qualities

Another question, raised by an anonymous reviewer, concerns what determines whether a portion of an abstract quality may relate to an individual. I take this question to ultimately relate to why the qualities discussed in this paper, such as love, hatred, and interest, possess targets, and therefore appear with target phrase arguments, but the qualities discussed by Francez and Koontz-Garboden, such as courage and beauty, do not.

In attempting an answer, I think it is instructive to consider similar variation in the domain of events. For example, running events may occur simply by virtue of there being a runner, while events of kicking may entail the existence of something kicked, or they may not, as when one moves one’s leg in a kicking motion. However, there can be no eating unless there is both someone doing the eating and something being eaten.

The domain of qualities can be broken down along similar lines: some qualities, such as courage or beauty, exist solely by virtue of being possessed, and are not directed at other individuals. Other qualities, such as sadness or anger, may be directed at certain things (my sadness at John’s death, my anger at Jim, etc.), but need not be. Finally, there are qualities, such as love and hatred, which, like eating, depend on two individuals for existence: they must be possessed by one individual, and directed at another in order to exist in the first place. This would put qualities on a par with other subsorts of individuals, and pave the way for a more fine-grained typology of qualities.

### Qualities vs. states

Lastly, there has been recent work on mental state and attitude verbs from the perspective of neo-Davidsonian event semantics, particularly Pasternak (2019), focusing on their gradability and corresponding measures of intensity. For example, Pasternak’s system explains the gradability of desire reports via an interaction between the *altitudes* of mental states *qua* eventualities and a measure function that is *monotonic* with respect to the part structure of those eventualities and their corresponding altitudes.

The work reported here has different explanatory goals from Pasternak’s: while Pasternak aims to explain the gradability of mental state verbs and a corresponding connection to the preference ordering at the heart of desire reports, my own concern has been with the expression of mental states via possessive predication and their semantic composition, with an analysis of modification and gradability as an additional boon of the analysis. Still, the choice between states and qualities is worth addressing, and I conclude the paper with a brief discussion of this issue.

As mentioned above, the measure functions at stake in the analysis of adjectives and mental state verbs as predicates of states, as in the work of Wellwood (2014) and Pasternak (2019), respectively, are required to be monotonic with respect to the parthood relation assumed for states, events, and ordinary individuals (Schwarzschild 2002). The relevant notion of monotonicity is defined in (136), and states that if s is a proper part of s’, then the measure of s must be strictly less than the measure of s’.

(136) s $<_{part}$ s’ → *μ*(s) < *μ*(s’)

The monotonicity of measurement explains why sentences like (137)may be interpreted as comparing the distance or temporal duration of the two events of running, but not their speed: distance and duration are monotonic measurements on running events, because a running event necessarily lasts longer than its parts, but speed is not, because the parts of a running event do not necessarily have lower speeds than the whole.

(137) John ran more than Mary (duration, distance, *speed)

Extending the claim to adjectives *qua* predicates of states and to mental state verbs amounts to the claim that measurements of, say, heat are monotonic with respect to hot states, and measures of intensity are monotonic with respect to mental states.

An issue arises for this approach, however: temporal duration should be monotonic on mental states as well. After all, intuitively, if I hate Mary from 1 pm to 3 pm, then there is a part of that state of hatred that lasts from 1 pm to 2 pm. And yet temporal duration does not seem to be available as a reading for comparatives with mental state predicates; the only reading available is one of intensity. This is true of English mental state verbs, and it holds true for Persian possessive experiencer complex predicates as well: (138)is not true in a context in which I have simply hated Ali for longer than I have hated Hasan, with no differences in the amount of hatred I have for them.^26^

(138)
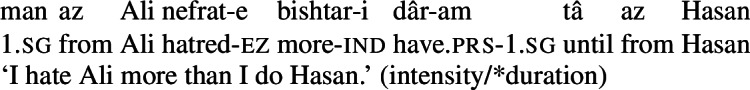

If temporal duration is not available with mental state comparisons, it must be because it is not a monotonic measurement on states. This would mean, given the definition of monotonicity in (123), that parts of states are not necessarily shorter in duration than the wholes of which they are part. That is to say, a part of a state of love or hatred (or, indeed, heat) may last just as long as a state of which it is part, but must be less intense than the larger state. This, however, makes mental states seem less like typical Davidsonian eventualities, the part structure of which is ordinarily intrinsically related to their spatiotemporal organization, and much more like portions of qualities, the part structure of which, on Francez and Koontz-Garboden’s approach as well as my own, is respected by their intrinsic size pre-order, but need not be connected to their temporal duration. In other words, the unavailability of temporal duration as a monotonic measurement in examples like (138)is *expected* on a quality possession analysis, but is not clearly so on the neo-Davidsonian state analysis. Though future work may provide a more in-depth comparison of the neo-Davidsonian and quality possession approaches, the discussion here at least provides some indication that the quality possession analysis is on the right track.

## Footnotes

1 Francez and Koontz-Garboden (2017) present a similar argument for investigating possessive predicating strategies for translational equivalents of adjectives in languages other than English. Francez and Koontz-Garboden (2017, p. 23) write: Languages vary in the extent to which they employ possessive and predicative strategies. For example, English and other Germanic and Romance languages employ both strategies, but the possessive one is very limited in scope, and in cases in which both exist with a single notional category, as in (i), the possessive member is very marked and, in many cases, unidiomatic.

2 Larson and Samiian (2020) refer to the three main dialect groups of Persian as iPersian, aPersian, and tPersian, respectively. As I do not compare different varieties of Persian in this paper, I do not make use of this terminology here.

3 *dâshtan* has an additional, unrelated use as a marker of progressive aspect (Taleghani 2010).

4 Persian is also able to express the standard of comparison with the preposition *az* ‘from.’ My consultants preferred the use of *tâ* here, in part due to the difficulty of processing sentences with both standard-marking *az* and the target prepositional phrase, itself sometimes headed by *az*.

5 Throughout this paper, I follow Francez and Koontz-Garboden (2017) in having variables p, p’, q, etc. range over portions of qualities. However, since these are more commonly used as variables over propositions in the formal semantics literature, to avoid confusion I explicitly type all variables with a type subscript, with variables over portions of type *e*, truth values of type *t*, and functions of type <*σ*,*τ*>, where *σ* and *τ* stand for any type. This departs slightly from the presentation in Francez and Koontz-Garboden (2017), who treat portions as of type *p*, with type *e* reserved for ordinary count and mass individuals. However, since they explicitly treat portions as subsorts of individual, I take this to be only a presentational departure, rather than one with serious technical implications.

6 In other words, ∃z^*I*^ requires that portions z be a member of I, and could alternatively be written as ∃z∈I. Likewise, ∃z$^{I\subset \text{\textsc{mind}}}$ could be expressed as ∃z∈I⊂mind, requiring z to be a member of a left-bounded interval, which is in turn a subset of the quality mind. I have chosen to maintain Francez and Koontz-Garboden’s notation here.

7 Francez and Koontz-Garboden are not explicit about how exactly the context saturates the interval argument. Where explicitness is required in what follows, I represent the contextually provided interval as an argument in the syntactic structure, and otherwise note that it is contextually provided.

8 There is another diagnostic for quality nouns, not directly applicable in English or Persian: quality nouns are known to be compatible with certain quantifiers in Italian, particularly *nessun(o)* ‘no,’ which are not compatible with ordinary mass or plural count nouns (Tovena 2001; Francez and Koontz-Garboden 2017; Zamparelli 2020).

*Nessun* is also compatible with mental state nouns, as (65)demonstrates with naturally occurring examples from the Web.

9 Recall that p is the type of portions for Francez and Koontz-Garboden (2017), a subsort of individual.

10 See, for example, Landman (2012) on the Unique Role Requirement for thematic roles, which states that thematic roles are functions from events to individuals. In other words, exactly one individual may bear any particular thematic role to an event.

11 Note that nothing changes if the meaning of the comparative is adjusted so that it denotes a relation between sets of intervals, an adjustment Francez and Koontz-Garboden themselves make to account for the equivalents of clausal comparatives in Ulwa. The two intervals will still be sets of portions with different targets, and are thus disjoint.

12 Both Li (2019) and Zhang (2020) propose extensions of Francez and Koontz-Garboden’s framework to deal with possessive predicating property concept sentences in Mandarin, which are gradable, and to differentiate them from other possessive constructions in the language, which are not. While these analyses are insightful in their own right, neither solves the problem of the direct participation of quality nouns in comparatives: on both analyses, gradability is introduced by the possessive verb, as in Francez and Koontz-Garboden’s approach.

13 A reviewer asks if target phrases permit other kinds of arguments, such as kind and quantificational noun phrases. The complement of the target preposition may indeed be a kind-denoting expression, as well as a quantified noun phrase, as the following two examples show.

I set aside kind and quantificational targets in the main text. That said, their properties are not really a problem for the analysis. For instance, if kinds are a type of individual (Carlson 1977; Chierchia 1998), the analysis I develop below for target phrases handles them straightforwardly. For quantified noun phrases, a range of solutions, such as Quantifier Raising (May 1985), may be adopted that permit interpretation of a quantificational argument in a position that expects an individual argument.

14 An alternative to the analysis in terms of function composition is one in which the subject is introduced below the target phrase, say in the specifier of the possessive VP, and undergoes raising to subject position in a higher specifier. This would saturate the possessor argument with a trace (interpreted as a type *e* variable), allowing the target phrase to compose with the VP, followed by abstraction over the variable, which is finally saturated by the possessor in subject position. I have chosen to use function composition in the main text largely because it enables composition to proceed *in situ*, but nothing hinges on this choice.

15 *C* can in fact be explicitly restricted to be a left-bounded interval of portions. The main difference from Francez and Koontz-Garboden’s approach, then, would be that *C* is not compositionally active.

16 I assume here a clausal analysis of *tâ* standards in Persian, and with it, a standard approach to the structure and interpretation of clausal standards, with operator movement, degree abstraction, and maximization over the resulting set of degrees (Kennedy 1999; Wellwood 2014). I further assume that the standard clause, while composing directly with *bishtar*, undergoes a process of right dislocation that accounts for its position to the right of the verb in the main clause of a comparative. Some evidence for a clausal analysis of *tâ* standards comes from the fact that the complement of *tâ* is not restricted to a single phrase, and may contain multiple constituents from the underlying clause, as in (90).

Such an example is difficult to accommodate on a phrasal approach to *tâ* standards, but is straightforwardly treated on the clausal analysis I adopt here. Still, the properties of comparative standards in Persian remain very much understudied in both the descriptive and formal literature on the language, and future research that sheds light on these properties would be valuable.

17 For reasons of space, I omit the compositional analysis of the standard clause, though see the previous footnote for a brief description of the analysis assumed.

18 Though I gloss *ziyâd* as ‘much’ in English, there are reasons for not assimilating it to the analysis of English *much* proposed in Wellwood (2014), on which it would simply denote a contextually provided measure function. For one, *ziyâd* is not used in the same range of constructions as *much*. For example, while English forms degree demonstratives and degree questions using *this/that/how much*, Persian uses *qadr* ‘amount’ to form such constructions.

In general, *ziyâd* is used more specifically to express large amounts, and is not readily modifiable. This is captured in the analysis proposed here, as *ziyâd*, unlike the comparative *bishtar*, introduces no degree argument, and simply requires the measure provided to exceed a standard.

19 This entailment to the positive does not occur with adjectival comparisons in Persian. (106), for instance, does not sound contradictory, as is generally expected of comparatives on, for instance, any degree-based analysis (Cresswell 1976; Kennedy 1999; Schwarzschild and Wilkinson 2002).

I have said nothing about the analysis of adjectives and adjectival comparisons in Persian in this paper, and I remain neutral with respect to their analysis. That said, given that nominal and VP comparisons make use of *bishtar*, and adjectival comparisons uniformly make use of the suffix *-tar*, it is not unreasonable to suppose that their semantic properties may be quite different. I thank an anonymous reviewer for asking about the entailments of comparatives with possessive experiencer complex predicates vs. adjectival comparisons.

20 I thank an anonymous NACIL3 reviewer for pointing this out to me, and for providing (108).

21 This is reminiscent of Zimmermann’s (1993) solution to the composition of quantificational noun phrases with intensional verbs, such as *seek*, which he analyzes as taking property arguments. For those quantificational NPs that cannot be converted into a property, the NP is instead scoped over the intensional VP, with an ident-shifted variable bound by the quantifier in the object position of the intensional verb. I am grateful to an anonymous reviewer for pointing out the relevance of Zimmermann’s analysis to this problem.

22 Many thanks to Saman Meihami and Baban Mohammed for contributing and discussing the Sorani Kurdish data here.

23 The dictionary is available online at www.focloir.ie, and provides example sentences to illustrate usage.

24 An alternative to this transparent analysis of the Irish sentences is to simply treat Irish as underlyingly structurally similar to Persian, but with syntactic conditions ruling out the pronunciation of the verb as *have*. This is essentially the approach taken in Noonan (1992) and Harves and Kayne (2012): for these authors, Irish lacks *have* as a lexical item capable of spelling out a possessive structure, and the prepositional phrases are present for Case-theoretic reasons. On this approach, there would be no underlying syntactic difference between Persian and Irish, and the analysis of Irish would be essentially the same as the analysis of Persian.

25 This analysis assumes that the existential verb *heje* serves to existentially close the portion variable, but nothing hinges on this. For instance, the verb may be treated as semantically vacuous, with an Existential Closure operation saturating the portion variable as in the analysis of Persian.

26 Pasternak is aware of this issue for his approach, and argues that, despite first impressions, temporal duration *is* available for mental state comparatives in the right context. He offers the following context (Pasternak 2019, pp. 294-295): For instance, imagine that some psychologists are running a study in which subjects are sent into house parties, where they witness various pleasant and unpleasant scripted events. The subjects are given a remote with a single button, and told to hold down the button whenever they decide they want to leave, and let go of the button whenever they stop wanting to leave. The remote does not provide any way of indicating how intense their desire to leave is. Now suppose that Subject A held the button from 7 pm to 10 pm, while Subject B only held the button from 6 to 6:30 and from 9:30 to 10. Thus, Subject A was uncomfortable for a total of three hours, while Subject B was uncomfortable for just one. In this scenario, it seems reasonable for a psychologist looking at the recorded results to report the following: (65) The results show that over the course of the evening, Subject A wanted to leave more than Subject B did. While Pasternak’s (65) may be felicitous in this context for some speakers (my own judgments are unclear), this context only establishes that *want* may permit temporal duration as a monotonic measure; similar contexts are difficult to construct for the mental states my own analysis is concerned with. Incidentally, Persian does not express desire reports using a possessive predicating strategy, but with the verb *xâstan*.

While the existence of a lexical verb for the expression of desire reports may simply be a coincidence, it is also possible that desire reports are not built on a quality possession semantics, which may explain their compatibility with temporal duration readings of comparatives.
